# Characteristics of Mussels-Derived Carbon Dots and Their Applications in Bio-Imaging and Detection of Riboflavin

**DOI:** 10.3390/foods11162451

**Published:** 2022-08-14

**Authors:** Wenyu Zhao, Yi Zhang, Bin Cao, Zhuoyan Li, Chengfeng Sun, Xiaolin Cao, Shuang Cong

**Affiliations:** College of Life Sciences, Yantai University, Yantai 264005, China

**Keywords:** carbon dot, mussel, green synthesis, fluorescent probe

## Abstract

A simple and green strategy has been demonstrated for the synthesis of carbon dots (CDs) from mussels. The chemical structure and optical properties of mussels-derived CDs prepared at different reaction temperatures (140, 160, and 180 °C) were evaluated. The average size of synthesized fluorescent CDs decreased from 2.06 to 1.30 nm as reaction temperatures increased from 140 to 180 °C. The fluorescence quantum yield of CDs could reach up to 15.20%. The surface of CDs was rich in functional groups such as -OH, -NH_2_, and -COOH, providing CDs with good water solubility and biocompatibility. Furthermore, the mussel-derived CDs have been successfully applied in bio-imaging for onion endothelium cells, HepG2 cells, and zebrafish. In addition, CDs could be employed as a biosensor for riboflavin detection. Therefore, mussels are a promising carbon resource for preparing N-doped CDs for bio-imaging and monitoring riboflavin.

## 1. Introduction

Carbon dots (CDs) are a kind of fluorescent carbon nanomaterial with a particle size of less than 10 nm [1]. They have been emerging as potential candidates for a variety of applications such as bio-imaging [2,3], sensors [4], and detection [5], owing to their superior properties, including good water solubility, excellent biocompatibility, chemical inertness, low cytotoxicity, easy functionalization, and anti-photobleaching [6]. Considering these outstanding properties and extensive applications, researchers have paid more attention to the fabrication of CDs.

In recent years, the preparation of CDs has been developed involving “top-down” and “bottom-up” methods [7], including chemical oxidation [8], microwave-assisted synthesis [9], laser irradiation [10], and so on. Due to the complicated equipment, low yield, and expensive raw materials required for preparing CDs, a green and convenient method of producing CDs is urgently needed. Among these methods, hydrothermal synthesis is an ideal approach, as it is simple, low cost, green, and produces a high fluorescence quantum yield [11,12]. Noteworthily, natural biomass is composed of biological macromolecules such as carbohydrates, proteins, and lipids, and contains elements such as nitrogen and sulfur, which could improve the fluorescence quantum yield of CDs without additional surface modifications [11,12,13]. Therefore, biomass including sweet lemon peels [13], mushrooms [14] durian shells, crab shells [15], cynostemma [16], carrots [17], etc., have been selected as precursors for preparing CDs via hydrothermal carbonization. The development of new biomass as a carbon source to produce CDs has received increasing attention [7].

Mussels are naturally inexpensive and rich in nutrients, which consist of proteins (58.7%), carbohydrates (22.5%), and lipids (7%). China has the highest production of mussels in the world, with an annual production of more than 800,000 tons [18]. Abundant components such as carbon, nitrogen, and oxygen motivate us to employ mussels as carbon sources to form cost-effective CDs with high quantum yields. Meanwhile, to the best of our knowledge, mussels have not been previously used to synthesize CDs.

In the current study, a facile, green, and low-cost method to prepare CDs derived from mussels is presented. The morphology properties, chemical composition, fluorescence properties, and stability of CDs formed at different reaction temperatures were investigated. Additionally, these fluorescent CDs were successfully applied to onion epidermal cells, HepG2 cells, and zebrafish for bio-imaging in vitro and vivo. Furthermore, mussels-derived CDs provided accurate detection of riboflavin.

## 2. Materials and Methods

### 2.1. Materials and Chemicals

Mussels were obtained from Xinshijie Supermarket (Yantai, China). HepG2 was supplied by China Center for Type Culture Collection in Wuhan, China. AB strain zebrafish (*Danio rerio*) were purchased from the Northern Center of the National Zebrafish Model Animals. High-glucose Dulbecco’s Modified Eagle’s Medium (DMEM) was supplied by GIBCO (Grand Island, NY, USA). All other chemicals and reagents were of analytical grade, bought from commercial companies unless otherwise stated.

### 2.2. Synthesis of Mussels-Derived CDs

Fresh mussel meat was washed with deionized water, homogenized, and freeze-dried for 24 h to obtain mussel powder. Then, 3 g of processed sample were added into 15 mL of deionized water and put in a polytetrafluoroethylene-lined hydrothermal synthesis kettle heated at 140, 160, and 180 °C for 8 h, respectively. After cooling to room temperature, the mixture was centrifuged (10,000 rpm, 10 min), and the supernatant was leached using ethyl acetate to remove oil phase. Subsequently, 4-folds absolute ethanol was added to the aqueous phase and placed at 4 °C for 24 h, then they were centrifuged (6000 rpm, 10 min, 4 °C) to remove polysaccharides. The supernatant was concentrated and purified with a D101 macroporous adsorption resin column using deionized water as an eluent by collecting the fluorescence fractions. The aqueous solution was concentrated followed by being dialyzed against distilled water with a membrane of the molecular weight of cutoff 500 Da for 24 h. Finally, the solution in the dialysis bag was filtrated with a 0.22 μm filtration membrane and lyophilized to obtain CD powder for future use. For the sake of description, CDs prepared at 140, 160, and 180 °C were named as 140-CDs, 160-CDs, and 180-CDs, respectively. Based on the dry weight of mussels, the yields of 140-CDs, 160-CDs, and 180-CDs were calculated to be 0.20%, 0.25%, and 0.56%, respectively.

### 2.3. Structural and Optical Characterizations of CDs

A transmission electron microscope (TEM, JEM-2100, JEOL, Tokyo, Japan) with an accelerating voltage of 200 kV was used to capture TEM images. Fourier-transform infrared spectroscopy (FT-IR, Frontier, PerkinElmer, Norwalk, CT, USA) was utilized to determine surface functional groups. Aromatic π-system analysis was conducted by UV-vis absorption spectrophotometer (TU-1900, Persee, Beijing, China). CDs crystallinity was studied using X-ray diffraction (XRD-6100, Shimadzu, Kyoto, Japan). X-ray photoelectron spectrometer (XPS, ESCALAB 250, Thermo VG, Waltham, MA, USA) was used to investigate elemental analysis.

Fluorescence spectroscopy was analyzed by a fluorescence spectrophotometer (RF-6000, Shimadzu, Kyoto, Japan). The fluorescence quantum yields [19] and time-resolved fluorescence spectra of CDs were determined according to previous studies [20].

### 2.4. Cytotoxicity and Bio-Imaging of CDs

An MTT assay was performed to analyze the cytotoxicity of 180-CDs at different concentrations (0, 0.375, 0.75, 1.5, 3, and 6 mg/mL) against HepG2 cells for 24 h [21].

Onion epidermal cells were chosen to investigate bio-imaging of CDs in plant cells. Briefly, a piece of onion epidermal membrane was soaked in 1.5 mg/mL of 180-CDs solution for 10 min, followed by being washed three times with saline. Then, a fluorescence microscope (DMi8, Leica, Wetzlar, Germany) with a 425 nm excitation wavelength was used to capture the images.

HepG2 cells are used in cell imaging. Briefly, HepG2 cells were incubated with 180-CDs for 24 h at 37 °C in a 5% CO_2_ incubator. After that, the cells were washed three times with phosphate buffer solution (PBS) and stored in PBS for optical imaging. The images were captured using a fluorescence inverted microscope (DMi8, Leica, Wetzlar, Germany) with a 425 nm excitation wavelength [22].

Zebrafish is transparent and served as an ideal model to evaluate the in vivo bio-imaging of CDs. Zebrafish larvae at 4 d were incubated in 1.5 mg/mL of 180-CDs solution and E3 medium without other substances as a control group for 48 h. An inverted microscope (DMi8, Leica, Wetzlar, Germany) with an excitation wavelength of 425 nm was used to observe the image.

### 2.5. Fluorescence Detection of Riboflavin by CDs

Briefly, different concentrations of riboflavin were added to CDs solution (0.075 mg/mL) within a Britton–Robinson buffer solution (BR, 200 mmol/L, pH 2.0), followed by reacting at room temperature for 10 min. Subsequently, the fluorescence spectrum of the mixture was measured, and fluorescence intensities at 537 nm and 437 nm were denoted as I_537 nm_ and I_437 nm_, respectively. Milk powder and riboflavin pharmaceutical tablets were chosen as the real sample. The pretreatment of actual samples was referred to in previous literature [23].

## 3. Results

### 3.1. Synthesis and Structural Characterizations of CDs

**Scheme 1:**
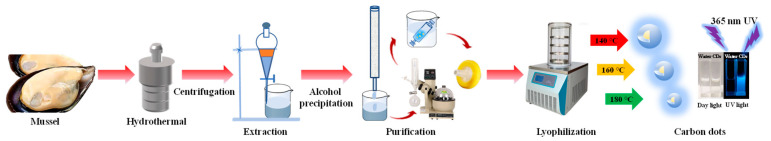
Synthesis of mussels-derived CDs by hydrothermal method at different reaction temperatures.

**Figure 1:**
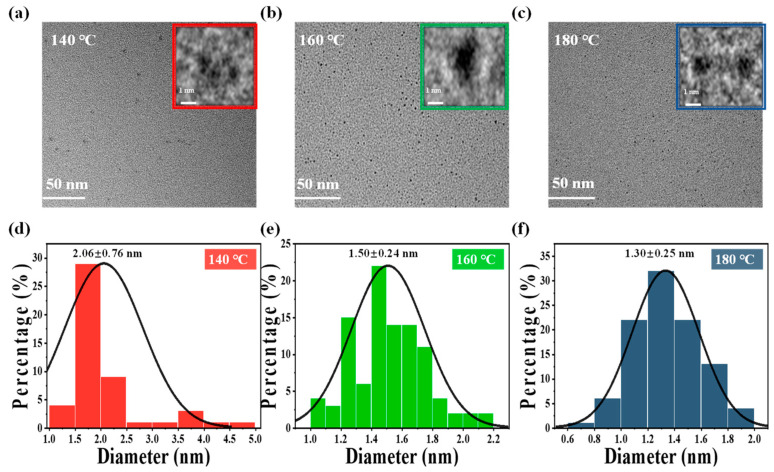
TEM images of CDs fabricated at (**a**) 140 °C, (**b**) 160 °C, (**c**) and 180 °C; insets represent high-resolution TEM images. The corresponding particle size distribution histogram of CDs formed at (**d**) 140 °C; (**e**) 160 °C; (**f**) 180 °C.

The fabrication of mussels-derived CDs is illustrated in Scheme 1. CDs were obtained using mussels as the carbon source through the hydrothermal synthesis process at 140, 160, and 180 °C for 8 h, respectively. TEM was applied to characterize the morphology of CDs (Figure 1), which indicated CDs were synthesized successfully and near-uniform spherical at different reaction temperatures. In addition, CDs prepared at 140 °C were slightly adhered to each other and aggregated, while CDs formed at higher temperatures were uniformly disperse (Figure 1a–c). It was observed that there was no significant crystal lattice in the CDs, even those prepared at 180 °C (Figure 1a–c insets). XRD patterns of CDs also displayed broad diffraction peaks at 2θ = 22.75°, 26.11°, and 25.34° (Figure S1), respectively, indicating the amorphous structure of CDs [22]. Meanwhile, the histogram in Figure 1d–f shows that mean size of the particles formed at 140, 160, and 180 °C were 2.06 ± 0.76 nm, 1.50 ± 0.24 nm, and 1.30 ± 0.25 nm, respectively. The enhanced carbonization at high temperatures as well as the high-temperature breakdown of polysaccharides and proteins in the mussel could contribute to the decrease in particle size with increasing temperature.

**Figure 2:**
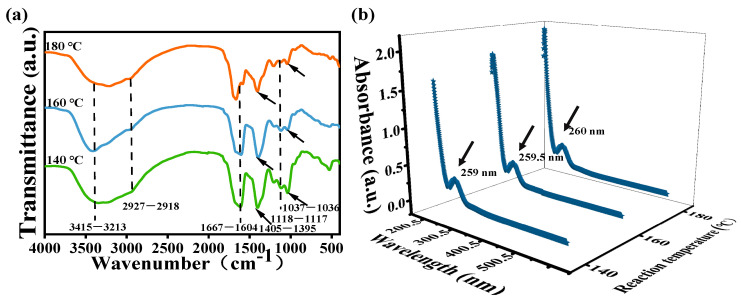
(**a**) FT-IR spectra; (**b**) UV–vis absorption spectra of mussels-derived CDs prepared at different reaction temperatures.

Furthermore, the functional groups of mussels-derived CDs produced at various reaction temperatures were determined by FT-IR and XPS. As instanced in Figure 2a, the broad absorption at 3415–3213 cm^−1^ could be ascribed to stretching vibrations of O–H or N–H. Peaks at 2918–2927cm^−1^ were identified as C–H stretching vibration. The strong band at 1667–1604 cm^−1^ was attributed to amide bonds (O=C-NH) or C=C in CDs [24,25,26]. The absorption peaks at 1405–1395 cm^−1^ were probably due to the C=O group [27]. In addition, the small peak around 1118 cm^−1^ was caused by the vibration of C–N [26]. The C–O bond is represented by the band at 1037 cm^−1^, which corresponded to the fingerprint area. FITR revealed that the surface of mussels-derived CDs contained a high variety of groups including -OH, C=O, and -NH_2_. In addition, the UV–vis spectrum presented apparently a strong absorption peak at 259–260 nm, indicating the π–π* transition of the C=C bonds of sp2 clusters from CDs [28] (Figure 2b).

**Figure 3:**
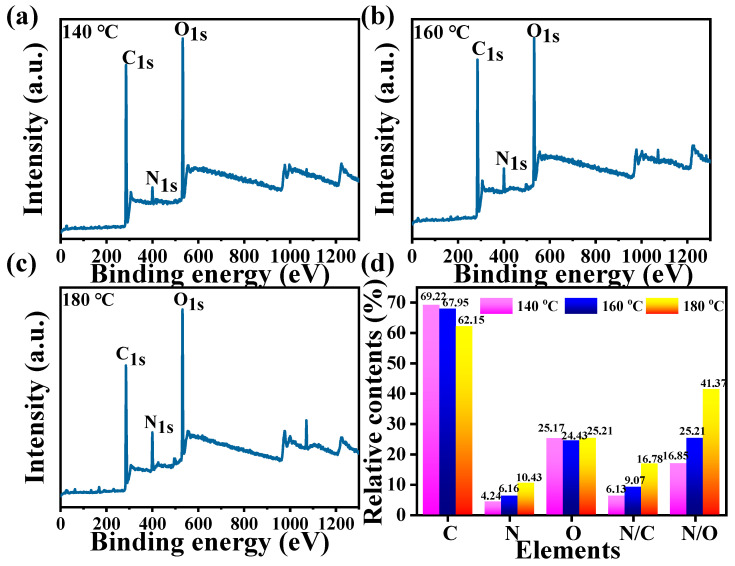
XPS spectra of mussels-derived CDs prepared at (**a**) 140 °C, (**b**) 160 °C, and (**c**) 180 °C, respectively; (**d**) relative contents of C, N, and O in mussels-derived CDs formed at different reaction temperatures.

**Figure 4:**
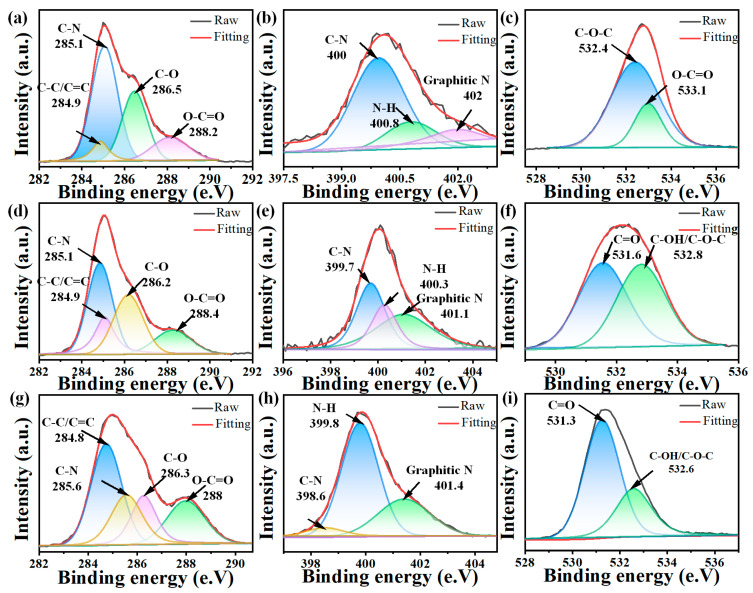
XPS high-resolution scans of the 140-CDs (**a**) C_1s_; (**b**) N_1s_, and (**c**) O_1s_. XPS high-resolution scans of the 160-CDs (**d**) C_1s_, (**e**) N_1s_, and (**f**) O_1s_. XPS high-resolution scans of the 180-CDs (**g**) C_1s_, (**h**) N_1s_, and (**i**) O_1s_.

XPS was used to determine the content of elements and accurate chemical bonds of CDs. As shown by the XPS survey spectrum, three predominant peaks at 285, 400, and 532 eV are assigned to C_1s_, N_1s_, and O_1s_, respectively (Figure 3a–c). The relative contents of C ranged from 69.22% to 62.15%, and the N content significantly increased from 4.24% to 10.43%, whereas there was no obvious change in the oxygen content (Figure 3d). Notably, the ratio of N/C and N/O increased by more than two-fold as the reaction temperature rose from 140 to 180 °C. This might be because the high temperature led more proteins of the mussels to be denatured and participate in the fabrication of CDs, which agrees well with previous studies [21]. What is more, the high-resolution XPS spectroscopy of C_1s_ of 140-CDs (Figure 4a) is represented by four peaks at 284.9, 285.1, 286.5, and 288.2 eV, which are linked to C=C or C–C, C=N, C=O, O=C-O, respectively [29]. The three peaks of N_1s_ at 400, 400.8, and 402 eV were attributed to C–N, N–H, and graphitic N (Figure 4b) [30]. Similarly, the same peaks or functional groups were present in the high-resolution spectra of C_1s_ and N_1s_ of 160-CDs and 180-CDs (Figure 4d,e,g,h). The O_1s_ spectrum of 140-CDs was deconvoluted into two peaks at 532.4 and 533.1 eV, which were imputed to C–O–C and C–OH or O=C-O, respectively (Figure 4c). Nevertheless, O_1s_ in 160-CDs and 180-CDs exhibited different binding energy peaks from 140-CDs. The two peaks were determined at 531.6 and 532.8 eV in 160-CDs while the peaks were 531.3 and 532.6 eV in 180-CDs, which corresponds to C=O and C–O/C–O–C [28]. The various chemical environments of O suggested that high temperatures decomposed macromolecules and created new chemical bonds in nanoparticles.

### 3.2. Optical Characteristics of CDs

**Figure 5:**
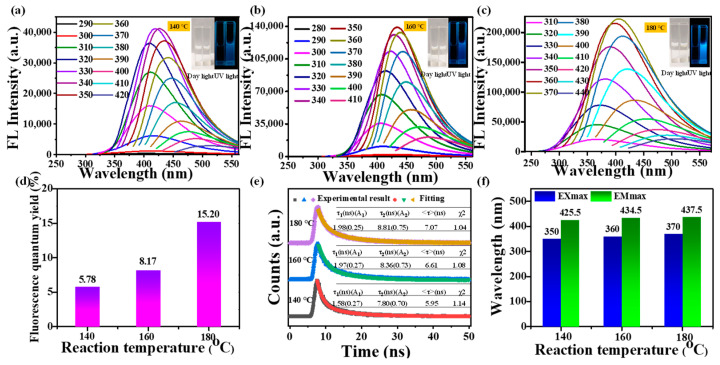
(**a**–**c**) Fluorescence emission spectra (the photographs of CDs water solutions under daylight and UV light were shown at the insets), (**d**) fluorescence quantum yield of mussels-derived CDs fabricated at 140 °C, 160 °C, and 180 °C, respectively, (**e**) fluorescence decay and fitting curves, (**f**) Ex_max_ and Em_max_ of mussels-derived CDs fabricated at 140 °C, 160 °C, and 180 °C, respectively.

The optical characteristics of CDs play a critical role in their bio-imaging application [30]. As seen from insets in Figure 5a, the aqueous solution of CDs (1 mg/mL) is transparent and almost colorless under daylight. The great water solubility of mussels-derived CDs is due to the presence of -OH, -C=O, and -NH_2_. In addition, CDs emitted blue fluorescence by a handheld laser lamp (365 nm), and CDs formed at higher reaction temperatures emitted stronger blue fluorescence at the same concentration. The fluorescence spectra of CDs presented a red shift of the emission wavelength with the increasing excitation wavelength (Figure 5a). The excitation-wavelength-dependent behavior could arise from diverse particle sizes or the existence of various emissive traps on the mussels-derived CDs’ surfaces [16]. As the reaction temperature increased, there was an obvious increase in the maximum excitation wavelength of mussels-derived CDs ranging from 350 to 370 nm, followed by maximum emission wavelengths changing from 425.5 to 437.5 nm (Figure 5b). This phenomenon is consistent with the reported results [21,31].

In addition, the average lifetime of CDs prepared at 140, 160, and 180 °C were measured to be 5.94, 6.61, and 7.07 ns, respectively, with fitted value χ^2^ < 1.2 (Figure 5c). The fluorescence lifetime was a reflection of the time it takes for the molecule to absorb energy and then return to the ground state [32]. The above results clearly confirm that the excited state of CDs was more stable along with the reaction temperature increasing. To confirm the differences in fluorescence intensity, the fluorescence quantum yield of mussels-derived CDs was evaluated using quinine sulfate as the standard substance (Figure S2). It was calculated that the fluorescence quantum yields of 140-CDs, 160-CDs, and 180-CDs were 5.78%, 8.17%, and 15.2%, respectively, consistent with the change in the CDs’ nitrogen content (Figure 5d). These phenomena indicated that more N in mussels was involved in the formation of CDs with the increasing reaction temperature, which was helpful to improve the fluorescence properties of CDs. This argument gains support from other natural biomass-derived CDs [33,34]. These observations further indicate reaction temperature is a key factor in the structural characteristics and optical properties of CDs formed by hydrothermal synthesis method.

### 3.3. Fluorescence Stability of Mussels-Derived CDs

**Figure 6:**
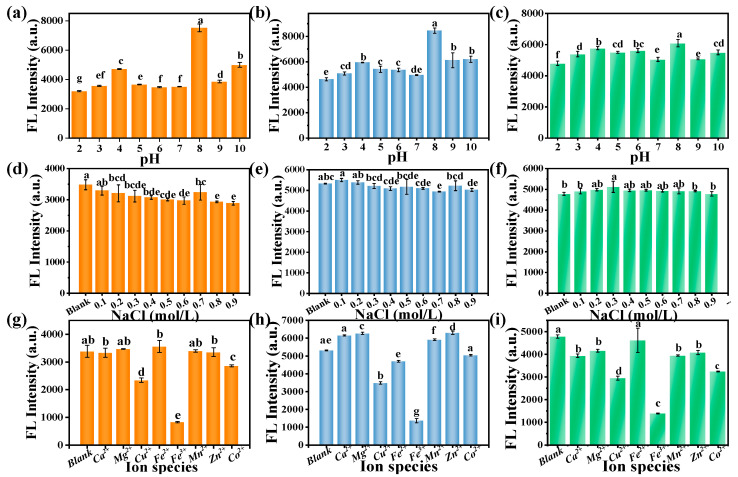
Effects of pH on the fluorescence (FL) intensity of (**a**) 140-CDs; (**b**) 160-CDs; (**c**) 180-CDs. Effects of the NaCl concentration on the fluorescence intensity of (**d**) 140-CDs; (**e**) 160-CDs; (**f**) 180-CDs. Effects of metal ions on the fluorescence intensity of (**g**) 140-CDs; (**h**) 160-CDs; (**i**) 180-CDs. Different lowercase letters (**a**–**g**) represent significant difference (*p* < 0.05).

In order to employ CDs for fluorescent probing, the fluorescence stability of CDs is a crucial prerequisite. Therefore, the effect of pH, NaCl concentration, and various metal ions on the CD fluorescence intensity was researched to evaluate the stability of mussels-derived CDs prepared at different reaction temperature (Figure 6). As illustrated in Figure 6a–c, the fluorescence intensity of CD solutions was sensitive to pH, the highest sensitivity occurring at pH = 8. The change in fluorescence behavior of CDs could be caused by the protonation as well as the deprotonation of functional groups (-OH, -NH_2_, and -COOH) on the surface of CDs [35]. Moreover, fluorescence intensity of 140-CDs dropped with the increasing concentrations of NaCl (Figure 6d) while there was no significant change in fluorescence intensity for 160-CDs or 180-CDs under different NaCl concentrations (Figure 6e,f). As for the effect of metal ions, it was worth noting that Cu^2+^ and Fe^3+^ had quenched the fluorescence intensity of mussels-derived CDs significantly. This could be attributed to the interaction between -NH_2_/-COO- on the surface of CDs and Fe^3+^ leading to the intense fluorescence quenching. The effect of Cu^2+^ quenching might be due to non-radiative electron transfer from the excited state of the Cu^2+^ electric orbital at the carbon site [36]. Consequently, all mussels-derived CDs have potential application as fluorescence sensors to quantitatively detect Fe^3+^, Cu^2+^ [37], and other components which could change their fluorescence.

### 3.4. Bioimaging of CDs in Onion Epidermal Cells and HepG2 Cells

**Figure 7:**
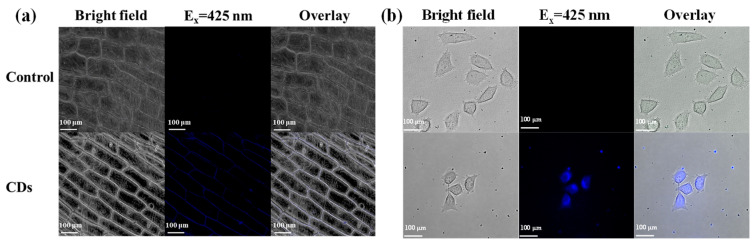
Image of (**a**) onion epidermal cells treated in 180-CDs for 10 min, (**b**) HepG2 cells treated with 180-CDs for 24 h. Cells without CDs treatment were used as control.

Taken together, the fluorescent CDs synthesized with mussels will be a potential fluorescent probe in the future. In this study, mussels-derived CDs prepared at 180 °C were used as bio-imaging and biosensor probes. Firstly, to estimate the biocompatibility of mussels-derived CDs, the viability of HepG2 cells treated with different concentrations of 180-CDs was evaluated. As shown in Figure S3, even though the concentration of 180-CDs increased to 6 mg/mL, the viability of HepG2 cells could still remain above 100% after incubation with CDs for 24 h. The result proved that mussels-derived CDs had excellent biocompatibility. Then, the 180-CDs were applied in onion epidermal cells and HepG2 cells for bio-imaging in vivo. Compared to the control, cell walls of the onion epidermal cells were fluorescent (Figure 7a), indicating that mussels-derived CDs could not cross the cytomembrane, which was consistent with pizza-derived CDs [38]. As seen in Figure 7b, HepG2 cells exhibited noticeable bright fluorescence after being treated with 180-CDs for 24 h, and mussels-derived CDs mainly localized in the cytoplasm of HepG2 cells. It was reported that endocytosis was the pathway for the cellular uptake of CDs [39]. Thus, mussels-derived CDs are excellent probes for cellular imaging applications.

### 3.5. In Vivo Imaging of Zebrafish

**Figure 8:**
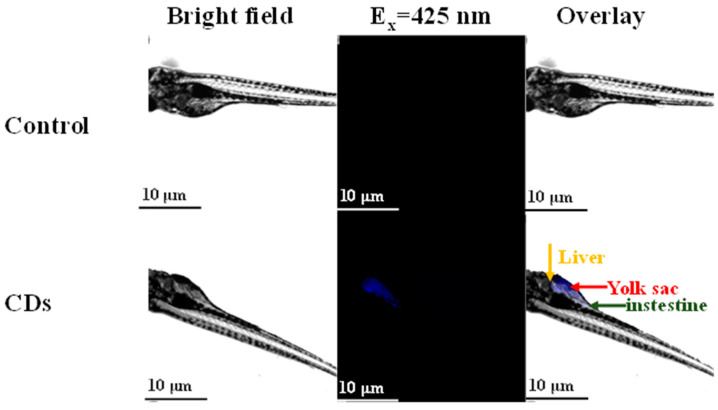
Bright field and fluorescence images of zebrafish larva incubated in 180-CDs for 48 h.

To assess the bioimaging ability of mussels-derived CDs in vivo, zebrafish larvae were fed with 180-CDs for 48 h. It was observed clearly that blue fluorescence was concentrated in the livers, yolks, and intestines of zebrafish incubated with CDs compared to the control group (Figure 8) [17]. This implies that CDs might enter into the zebrafish by mouth. These results signify that mussels-derived CDs are biocompatible and appropriate for bio-imaging in vivo.

### 3.6. Application of CDs for Riboflavin Detection

#### 3.6.1. Optimization of Riboflavin Detection Conditions

Riboflavin, i.e., vitamin B2, plays a crucial role in keeping organism health, and their content could also infer food quality; therefore, it is of great importance to provide accurate riboflavin analytical methods. It is worth noting that CDs could be employed as a ratio-metric sensor for riboflavin based on fluorescence resonance energy transfer (FRET) [40]. To confirm the potential for FRET between mussels-derived CDs (donor) and riboflavin (acceptor), the UV-vis absorption spectrum of riboflavin was measured. As illustrated in Figure S4, riboflavin produced four strong absorbance peaks at 200, 260, 370, and 450 nm. Meanwhile, there was an overlap region between the absorption spectrum of riboflavin and the emission spectrum of mussels-derived CDs, which indicates the potential for FRET between mussels-derived CDs and riboflavin [40]. Before investigation of CD detection performance for riboflavin, the effects of pH and reaction time on riboflavin detection were examined. As shown in Figure S5, the ratio I_537 nm_/I_437 nm_ of the CDs/riboflavin system achieved its highest level after reacting for 10 min, then fell slightly, owing to riboflavin degraded to lumiflavin [40]. Moreover, the I_537 nm_/I_437 nm_ ratio decreased with pH increasing because the hydroxyl and carboxyl groups on the surface of CDs were protonated while riboflavin was relatively stable at low pH [41]. Therefore, the optimum reaction time and pH for measurement were identified as 10 min and 2, respectively.

#### 3.6.2. Analytical Performance of CDs in the Detection of Riboflavin

**Figure 9:**
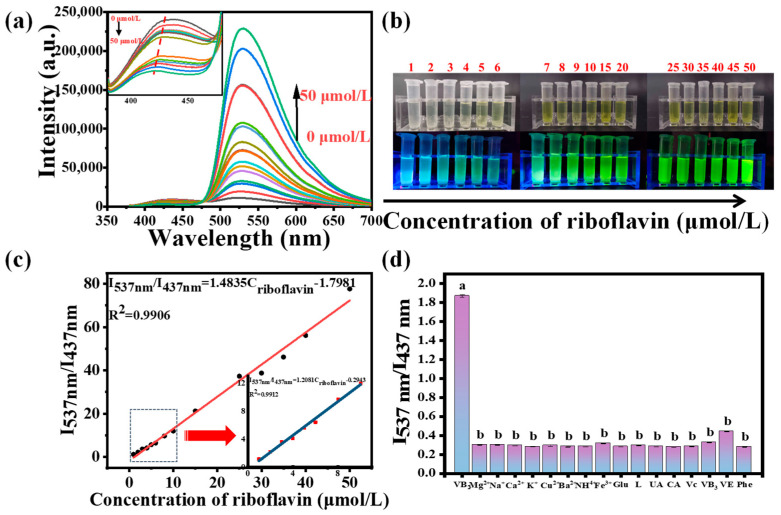
(**a**) Fluorescence emission spectra and (**b**) photographs of CDs–riboflavin system under 365 nm UV light during stepwise titration of riboflavin with 0–50 μmol/L, (**c**) linear relationship between I_537 nm_/I_437 nm_ and the concentration of riboflavin in the range 1–50 μmol/L, (**d**) the selectivity of the proposed method for riboflavin under optimal experimental conditions. Different lowercase letters (**a**,**b**) represent significant difference (*p* < 0.05).

After optimization of riboflavin assay conditions, quantitative detection of riboflavin was evaluated under optimal experimental conditions. As seen in Figure 9a, the fluorescence spectra and fluorescence intensity of CDs at 437 nm progressively blue-shifted and reduced, respectively, while the fluorescence intensity of riboflavin at 537 nm steadily rose with the addition of riboflavin. This phenomenon was consistent with the photographs of the CDs–riboflavin system under UV light (365 nm) (Figure 9b). According to the FRET efficiency equation (η = 1 − I/I_0_, where I and I_0_ correspond to the fluorescence intensity of the CDs at 437 nm and 537 nm in the presence and absence of riboflavin), the efficiency increased from 0% to 67.2% while the concentration of riboflavin increased from 0 to 50 μmol/L. In addition, there was a good linear relationship between I_537 nm_/I_437 nm_ and the concentration of riboflavin in the range of 1–50 μmol/L (Figure 9c): I_537 nm_/I_437 nm_ = 1.4835 C_riboflavin_ − 1.7981, and R^2^ = 0.9906. Similarly, I_537_ nm/I_437_ nm also showed a good linear relationship with the concentration of riboflavin in the range 1–10 μmol/L (Figure 9c inset). The limit of detection was calculated to be 6.06 nmol/L based on 3S_b_/k. Compared with other sensors, the proposed strategy has a wide linear range and relatively low detection limit (Table 1).

Furthermore, the selectivity of the sensing system was investigated using common metal salt solutions, amino acids, and food additives at a concentration of 95 umol/L. As shown in Figure 9d, a significant change in the fluorescence intensity ratio (I_537 nm_/I_437 nm_) was only discovered in the presence of riboflavin. The reasons are as follows. Firstly, mussels-derived CDs had a good fluorescence stability against these analytes whose concentration was 95 umol/L, which caused a relatively stable fluorescence intensity at 437 nm and 537 nm. Moreover, the riboflavin has a strong fluorescence intensity at 537 nm and could quench the fluorescence of CDs for FRET. These results indicated that the developed ratiometric fluorescence biosensor can selectively detect riboflavin.

#### 3.6.3. Analysis of Riboflavin in Real Samples

A practical application of the approach was employed to detect riboflavin in milk, milk powder, and riboflavin pharmaceutical tablets. A recovery experiment was further conducted by adding different concentrations of riboflavin into these samples. As shown in Table 2, the recoveries ranged from 93.0% to 101.0%, and a relative standard deviation was 0.4 to 1.3%, which illustrated the feasibility of the method to detect riboflavin in real samples.

## 4. Conclusions

In conclusion, mussels are a promising carbon resource for preparing N-doped CDs with good fluorescence quantum yield up to 15.20% via a green fabrication method. These mussels-derived CDs had a narrow size range, and the minimum average particle size was 1.30 nm. The obtained CDs contained C, N, O, and H and rich functional groups such as -OH, -NH_2_, and -COOH on their surface. These CDs exhibited excellent water solubility and bright blue fluorescence. Moreover, mussels-derived CDs were successfully employed as a probe for bio-imaging in vitro and in vivo for their good fluorescence stability and biocompatibility. Furthermore, CDs were applied to the quantitative detection of riboflavin. Overall, mussels-derived CDs are expected to be developed as fluorescence probes for bio-imaging and food ingredients or contaminants detection. These results provided important insights into the preparation, characteristics, and application of CDs derived from natural biomass.

## Figures and Tables

**Table 1:** Comparison of different sensors for the detection of riboflavin.

Sensors	Linear Range (μmol/L)	Detection Limit (nmol/L)	Reference
N, S-CDs	0.56–7.44	1.9	[40]
g-C3N4 nanosheets ^1^	0.4–10	170	[42]
Ag nanoparticles	0.17–4.67	3.7	[43]
N, P-CDs	0.5–50	170	[23]
g-CNQDs@Zn-MOF ^2^	0.005–1.0	15	[44]
Mussels-derived CDs	1–50	6.06	This work

^1^ Graphitic carbon nitride nanosheets. ^2^ Graphitic carbon nitrides quantum dots-Zn-MOF composite.

**Table 2:** Detection of riboflavin in real samples with the proposed method.

Sample	Measured (μmol/L)	Added (μmol/L)	Found (μmol/L)	Recovery (%)	RSD (%)
Milk	0.45	40.00	40.50	100.1	1.3
		20.00	20.41	99.8	0.7
		4.00	4.38	98.3	1.1
Milk powder	0.98	40.00	38.18	93.0	0.7
		20.00	19.98	95.0	0.5
		4.00	4.87	97.3	1.2
Riboflavin tablet	5.12	40.00	45.52	101.0	0.9
		20.00	24.99	99.4	0.6
		4.00	9.05	98.3	0.4

## Data Availability

The datasets generated for this study are available on request to the corresponding author.

## Supplementary Materials

The following supporting information can be downloaded at: https://www.mdpi.com/article/10.3390/foods11162451/s1, Figure S1: XRD pattern of mussels-derived CDs. Figure S2: Fluorescence quantum yield of quinine sulfate and mussels-derived CDs prepared at (a) 140 °C; (b) 160 °C, and (c) 180 °C. Figure S3: Cell viability of HepG2 cells after incubation with different concentrations of CDs for 24 h. Figure S4: UV-vis absorption spectrum of riboflavin and fluorescence emission spectrum of 180-CDs. Figure S5: (a) Effects of the reaction time, and (b) pH on the detection of riboflavin between CDs and riboflavin.
