# Supplementary material for: Characteristics of Mussels-Derived Carbon Dots and Their Applications in Bio-Imaging and Detection of Riboflavin
Source: Foods. 2022 Aug 14;11(16):2451. doi: 10.3390/foods11162451 (PMC9407624; doi:10.3390/foods11162451)
Supplement: Supplementary file 1 [file foods-11-02451-s001.zip › foods-1797966-supplementary.pdf]

Characteristics of mussels-derived carbon dots and their applications in bio-imaging and detection of riboflavin

Wenyu Zhao, Yi Zhang, Bin Cao, Zhuoyan Li, Chengfeng Sun, Xiaolin Cao, Shuang Cong*

College of Life Sciences, Yantai University, Yantai 264005, Shandong, China

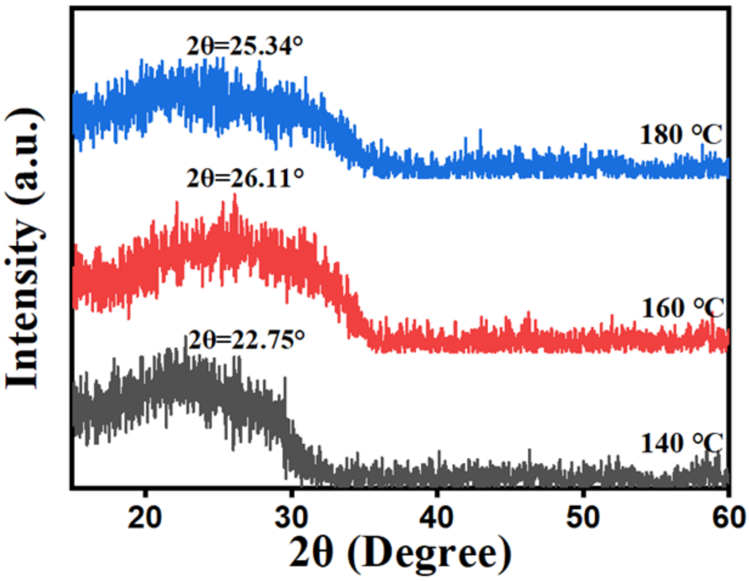

Figure S1. XRD pattern of mussels-derived CDs.

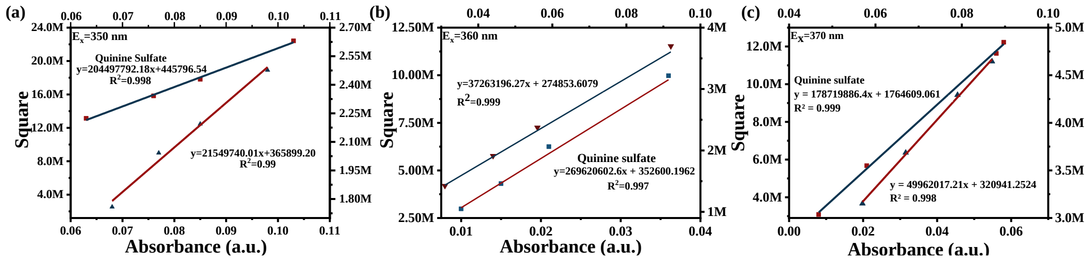

Figure S2. Fluorescence quantum yield of quinine sulfate and mussels-derived CDs prepared at (a) 140 °C, (b) 160 °C, and (c) 180 °C.

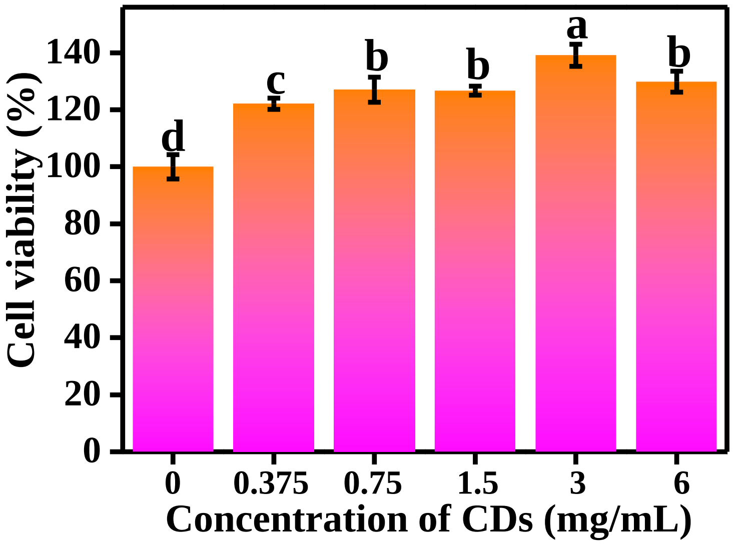

Figure S3. Cell viability of HepG2 cells after incubation with different concentrations of CDs for 24 h. Different lowercase letters (a–d) represent significant difference ($p < 0.05$).

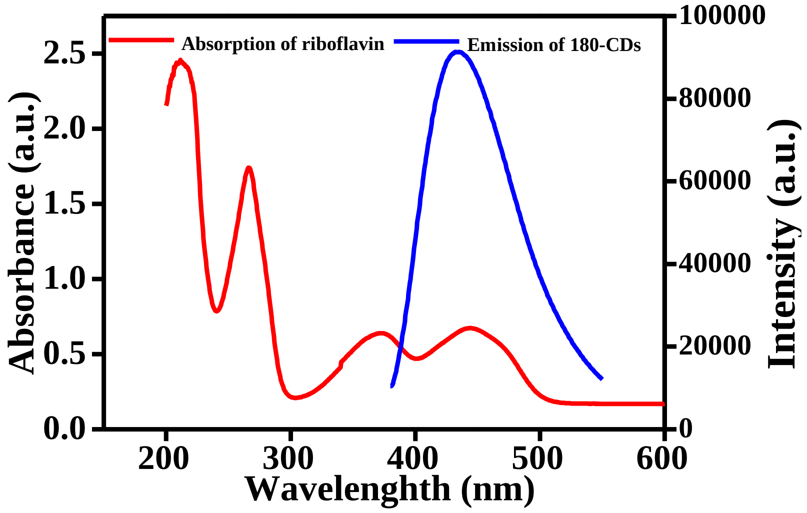

Figure S4. UV-vis absorption spectrum of riboflavin and fluorescence emission spectrum of 180-CDs.

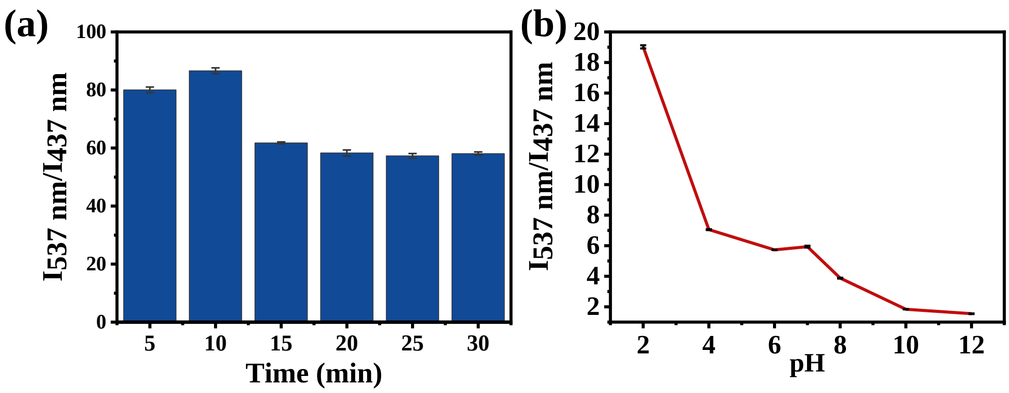

Figure S5. (a) Effects of the reaction time, and (b) pH on the detection of riboflavin between CDs and riboflavin.
